# Tracking preleukemic cells in vivo to reveal the sequence of molecular events in radiation leukemogenesis

**DOI:** 10.1038/s41375-018-0085-1

**Published:** 2018-03-03

**Authors:** Tom Verbiest, Rosemary Finnon, Natalie Brown, Lourdes Cruz-Garcia, Paul Finnon, Grainne O’Brien, Eleanor Ross, Simon Bouffler, Cheryl L. Scudamore, Christophe Badie

**Affiliations:** 1 Cancer Mechanisms and Biomarkers Group, Radiation Effects Department, Centre for Radiation, Chemical and Environmental Hazards, Public Health England, Didcot, OX11 ORQ UK; 2 CRUK & MRC Oxford Institute for Radiation Oncology, Department of Oncology, University of Oxford, Oxford, OX3 7DQ UK; 3 Mary Lyon Centre, MRC Harwell, Oxfordshire, OX11 0RD UK

## Abstract

Epidemiological studies have demonstrated an increased leukemia incidence following ionizing radiation exposure, but to date, the target cells and underlying mechanisms of radiation leukemogenesis remain largely unidentified. We engineered a mouse model carrying a different fluorescent marker on each chromosome 2, located inside the minimum deleted region occurring after radiation exposure and recognized as the first leukemogenic event. Using this tailored model, we report that following radiation exposure, more than half of asymptomatic CBA *Sfpi1*^*GFP/mCh*^ mice presented with expanding clones of preleukemic hematopoietic cells harboring a hemizygous interstitial deletion of chromosome 2. Moreover, following isolation of preleukemic hematopoietic stem and progenitor cells irradiated in their native microenvironment, we identified the presence of *Sfpi1* point mutations within a subpopulation of these preleukemic cells expanding rapidly (increasing from 6% to 55% in 21 days in peripheral blood in one case), hence identifying for the first time the presence of such cells within a living animal. Importantly, we also report a previously undescribed gender difference in the phenotype of the preleukemic cells and leukemia, suggesting a gender imbalance in the radiation-induced leukemic target cell. In conclusion, we provide novel insights into the sequence of molecular events occurring during the (radiation-induced) leukemic clonal evolution.

## Introduction

It is widely recognized that exposure to ionizing radiation increases leukemia incidence [1–6]. Radiation leukemogenesis is a genetically complex, multistep process, and the underlying mechanisms and target cells remain unidentified [7]. The CBA inbred mouse strain is a model of radiation-induced acute myeloid leukemia (rAML) [8, 9] where hemizygous interstitial deletion of chromosome 2 (Del2) is a characteristic finding [10] with the minimal deleted region (MDR) containing *Sfpi1*, encoding the hematopoietic transcription factor PU.1 [11]. In ~85% of the cases, the remaining *Sfpi1* copy carries a point mutation in a single CGC codon, within the DNA binding domain in exon 5 [12]. These biallelic *Sfpi1* aberrations support a two-hit model in murine rAML [13]. Bone marrow (BM) cells carrying Del2 can be identified 24 h post-irradiation, and it is assumed that Del2 HSPCs expand clonally [14]. Ultimately, 15-20% of mice will present with AML [15].

All data generated previously used fixed leukemic cells to study Del2 and *Sfpi1* mutations, thus limiting further characterization of leukemogenesis. Here, we crossed CBA *Sfpi1*^*GFP/GFP*^ mice [16] with a newly generated CBA *Sfpi1*^*mCh/mCh*^ transgenic model to create an F1 CBA *Sfpi1*^*mCh/GFP*^ mouse expressing mCherry from a Rosa26 promotor construct located in the chromosome 2 MDR, and GFP being expressed from the other allele under the *Sfpi1* promoter. Monthly blood sampling post-irradiation was used to monitor Del2, and preleukemic clonal expansion, by flow cytometry.

We report that more than half of mice presented with preleukemic cells harboring Del2. Moreover, we identified for the first time the presence of *Sfpi1* point mutations within subpopulations of these preleukemic cells, within a living animal. We also provide evidence of a gender difference in the (pre)leukemic phenotype, suggesting a difference in the leukemic target cell between male and female mice.

## Methods

### Mice, rAML induction, and tissue preparation

CBA *Sfpi1*^mCh/mCh^ mice were generated as previously described [17], and mated to CBA *Sfpi1*^GFP/GFP^ mice [16] to generate F1 CBA *Sfpi1*^mCh/GFP^ mice. Mice were given single 3Gy whole-body X-irradiation at 10-12 weeks of age (70 males and 50 females). Sham-irradiation of age-matched mice (*n* = 20; assigned at random) was performed by placing the mice into the irradiator box for the appropriate time without X-rays being produced. rAMLs were diagnosed as described previously [16], using the criteria of the ‘Bethesda proposals for classification of nonlymphoid neoplasms in mice’ [18]. Spleen tissue was stored at −70 °C in RNA*later*® (Ambion, Austin, US) for nucleic acid extraction, in 4% formaldehyde for histopathological analysis, or disaggregated for FACS analysis, as described previously [16]. All animal procedures conformed to the UK Animals (Scientific Procedures) Act, 1986, Amendment Regulations 2012, and animal experimental protocols were reviewed and approved by the local Ethics Committee and the Home Office.

### Immunophenotyping of leukemic spleen cells

Spleen cells were incubated with phycoerythrin (PE)-conjugated antibodies: Sca1 (E13-161.7; BioLegend), cKit (2B8; Abcam, Cambridge, UK), Flt3 (A2F10.1), Gr1 (1A8), Ly6c (HK1.4; Abcam), Mac1 (M1/70), CD31 (MEC13.3), CD3 (17A2), and B220 (RA3-6B2). All reagents were purchased from BD Biosciences, unless otherwise stated. Acquisition was performed using a Guava® easyCyte Single Sample flow cytometer, and analyzed using InCyte™ software (Merck Millipore, Watford, UK).

### DNA isolation from spleens and sequencing for Sfpi1 exon 5 point mutation

DNA was extracted from spleen tissue using a DNeasy® Blood & Tissue kit (Qiagen, Manchester, UK). Exon 5 mutations in *Sfpi1* were determined by DNA sequencing as described previously [19, 20], using primer sequences forward 5′-CGACATGAAGGACAGCATCT-3′ and reverse 5′-TTTCTTCACCTCGCCTGTCT-3′ (IDT, Leuven, Belgium).

### PCR for mCherry and GFP construct detection

Detection of GFP construct was performed as previously described [16]. For detection of the mCherry construct, primer sequences were Cel1-F 5′-GTGACTCCCAACATCTGCCT-3′, Cel1-R 5′-CTGCTTGCTTGCAGACTGAG-3′, Donor-F3 5′-AAGGGCGAGGAGGATAACAT-3′ and Donor-R3 5′-CTTCAGCTTCAGCCTCTGCT-3′ (IDT).

### Immunomagnetic cell separation and fluorescence-activated cell sorting

Lin− cells were selected using EasySep™ Mouse Hematopoietic Progenitor Cell Enrichment Kit (Stem Cell Technologies, Grenoble, France) and incubated with the following antibodies conjugated with PE, PE-Cy5, PE-Cy7, fluorescein isothiocyanate (FITC), allophycocyanin (APC) or APCeFluor®780: Sca1 (D7), cKit (2B8), CD48 (HM48-1), CD127 (A7R34), and CD150 (TC15-12F12.2; BioLegend, San Diego, USA). All reagents were purchased from Affymetrix (High Wycombe, UK), unless otherwise stated. Flow cytometry acquisition and sorting was performed using MoFlo XDP (Beckman Coulter, High Wycombe, UK).

### Pyrosequencing analysis

DNA was extracted from blood (10 µl) using DNeasy® kit (Qiagen). Ten ng of DNA was used to amplify the target sequence of *Sfpi1* exon 5 by PCR with primer biotinylated forward 5′-GCATCCAGAAGGGCAACC-3′ and reverse 5′-TCGCCTGTCTTGCCGTAGT-3′ primers generating a 79 bp PCR product. Primers, DNA and PyroMark PCR master mix (Qiagen) were combined in a total volume of 25 µl, and amplified: 15 min at 95 °C, then 45 cycles of (30 s at 95 °C, 30 s at 60 °C and 30 s at 72 °C). Ten µl of the biotinylated PCR product was used to detect mutations on the first base of the 235th codon (CGC to TGC) with the following sequencing primer: 5′-CCTGTCTTGCCGTAGT-3′ using PyroMark48 (Qiagen).

## Results

### Clonal expansion of Del2 hematopoietic cells following radiation exposure

Mice received 3 Gy whole-body X-irradiation and underwent monthly tail vein bleeding for lifespan to identify leukocytes carrying Del2, indicated by fluorescence loss (Supplementary Figure S1A). In sham-irradiated mice, all leukocytes expressed mCherry. GFP expression is controlled by PU.1 promoter, hence lymphocytes did not express GFP (i.e., mCherry+GFP−). Myeloid cells (monocytes and granulocytes) require PU.1 for terminal differentiation and maturation, and expressed GFP throughout lifespan (i.e., mCherry+GFP+). Either PU.1 copy (mCherry or GFP) can be deleted during leukemic transformation. However, mCherry loss was used as the ‘lead’ for detection of Del2, allowing GFP to be used as a proxy for PU.1 expression from the remaining copy.

**Fig. 1:**
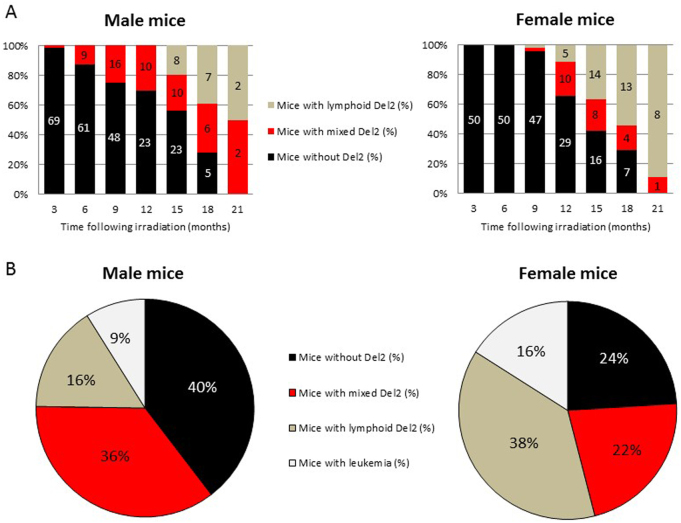
Clonal expansion of mCherry− leukocytes in peripheral blood. Blood of irradiated male and female CBA *Sfpi1*^mCh/GFP^ mice (*n* = 70 and *n* = 50, respectively) was analyzed monthly for mCherry and GFP expression. **a** Percentage of male (left panel) and female (right panel) mice without mCherry loss (black), with both myeloid and lymphoid mCherry loss (red), and with lymphoid mCherry loss (green), detected in the blood, as a function of time following radiation exposure. Number in the bar reflects the actual number of animals alive at the time point. **b** Percentage of male (left panel) and female (right panel) mice at time of death, diagnosed without mCherry loss (black), with both myeloid and lymphoid mCherry loss (red), with lymphoid mCherry loss (green) or with leukemia (white)

In irradiated animals, clonal expansion of mCherry negative (mCherry−) leukocytes was detected as early as 3 months post-irradiation (Fig. 1a). At 9 months, the percentage of male mice with mCherry− leukocytes was markedly higher compared with females (25% and 4%, respectively), increasing to 70% for both at 18 months (Fig. 1a). Interestingly, the percentage of female mice with clonal expansion of only mCherry− lymphocytes was markedly higher compared with males (54% and 39%, respectively) at 18 months, and increased further by 21 months (Fig. 1a). At death, the percentage of male mice with no mCherry− leukocytes (40% and 24%, respectively), as well as mCherry− mixed myeloid–lymphoid leukocytes was higher than in females (36% and 22%, respectively; Fig. 1b). Importantly, almost twice as many females were diagnosed with leukemia compared with males (16% and 9%, respectively; Fig. 1b). Irradiated mice with Del2 clonal expansion and unirradiated mice had similar WBC counts (6.8 ± 3.4 × 10^6^/ml and 7.9 ± 2.6 × 10^6^/ml, respectively). Only at the time of overt leukemia presentation, WBC counts were increased (92.7 ± 121.8 × 10^6^/ml).

### Altered PU.1 expression in Del2 leukocytes and hematopoietic progenitors

As most male mice presented with clonal expansion in mCherry− myeloid–lymphoid leukocytes, we hypothesized that Del2 occurred in a very primitive hematopoietic cell type: hematopoietic stem cell (HSC) or multipotent progenitor (MPP). To further characterize the cell of origin, three irradiated male mice with mCherry− myeloid–lymphoid leukocytes were killed. Flow cytometry analysis showed that about 10% Lin− cells were mCherry−. Approximately 0.8% of mCherry−Lin− cells were Sca1+cKit+, and half of the mCherry− LSK fraction was CD48-CD150+ (i.e., LT-HSCs/MPP1), indicating that these HSCs carried Del2 (Supplementary Figure S2A). Similarly, mCherry− myeloid (i.e., mCherry−Lin−Sca1−cKit+) and lymphoid progenitors (i.e., mCherry−Lin−CD127+) were identified (Supplementary Figure S2B).

Interestingly, at 18 months, 54% of female mice had clonal expansion of only mCherry− lymphocytes. Peripheral blood analysis showed that ±50% of lymphocytes were mCherry−. However, all monocytes and granulocytes were mCherry+ (Supplementary Figure S3B). BM analysis revealed that only 1.9% of Lin− cells were mCherry−, compared with 10% in mice with clonal expansion in mCherry− myeloid–lymphoid leukocytes. Almost all mCherry−Lin− cells were Sca1−cKit− (96%). Although the cell number was low, in these samples no mCherry− HSCs or mCherry− myeloid progenitors could be identified (Supplementary Figure S3A). However, 11% of mCherry−Lin− cells expressed CD127, indicating that Del2 most likely occurred in an immature lymphoid cell type (CLP; Supplementary Figure S3B).

**Fig. 2:**
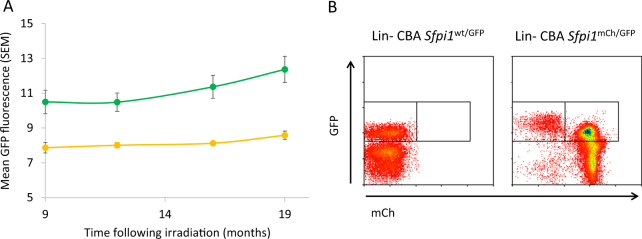
GFP expression is upregulated in myeloid leukocytes following Del2. **a** Blood of 8 male mice with clonal expansion in mCherry− leukocytes was analyzed for mCherry and GFP expression. Mean GFP fluorescence in mCherry+ and mCherry− granulocytes was compared over time. Error bars indicate SEM. **b** Representative plots of GFP expression in Lin− cells from a sham-irradiated male CBA *Sfpi1*^wt/GFP^ mouse, and an irradiated male CBA *Sfpi1*^mCh/GFP^ mouse with clonal expansion in mCherry− leukocytes (left and right panel, respectively). GFP expression was higher in mCherry−Lin− cells, compared with mCherry+Lin− cells

To determine PU.1 expression changes on the remaining chr2 after exposure, blood of male mice with clonal expansion of mCherry− leukocytes was analyzed for mCherry/GFP expression. When comparing GFP expression between mCherry− and mCherry+granulocytes, mCherry− granulocytes had a markedly higher GFP expression (Fig. 2a). Similarly, GFP expression was higher in mCherry−Lin− than in mCherry+Lin− cells (Fig. 2b).

### Exon 5 Sfpi1 point mutation is solely observed in Del2 hematopoietic cells

**Fig. 3:**
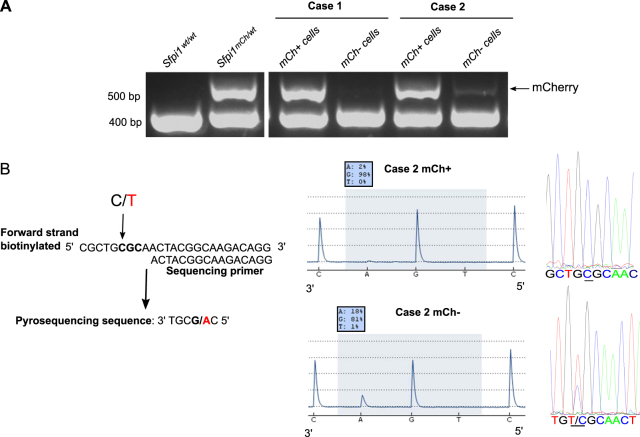
Exon 5 *Sfpi1* point mutation is solely observed in Del2 hematopoietic cells. **a** Lin− cells of male mice were sorted based on mCherry expression. Absence of mCherry in mCherry−Lin− cells was demonstrated [Rosa26-mCherry allele (510 bp); wild-type allele (383 bp)]. **b** Schematic representation of sequence analyzed by pyrosequencing using sequencing primer complementary to biotinylated forward strand. Pyrograms depict allele quantification for the first base of the 235th codon of the *Sfpi1* gene for mCherry+Lin− and mCherry−Lin− cells. Pyrograms indicate the complementary sequence to the forward strand, therefore the % of **a** indicates the lack or presence of the mutation. Sanger sequencing profiles are presented as well for the same mCherry+Lin− and mCherry−Lin− cells

There are data suggesting that the *Sfpi1* point mutation most probably leads to complete abolition of PU.1 activity [21, 22]. To identify and quantify the presence of the ‘second’ driving mutation, Lin− cells were sorted, based on their mCherry expression, from irradiated male mice with mCherry− blood leukocytes. mCherry allele loss occurred specifically in the sorted mCherry− population (Fig. 3a). Overall, 16% of mCherry−Lin− cells in mouse 2 had the characteristic murine rAML C to T substitution, indicating that these mutations are restricted to Del2 Lin− cells (Fig. 3b). Point mutations were also analyzed in terminal blood samples. Although not detectable in sham or irradiated CBA *Sfpi1*^mCh/GFP^ mice with no mCherry− leukocytes, 83% of irradiated mice harboring mCherry− clonal expansion showed detectable levels of C to T substitution, linking point mutation occurrence to a prior presence of Del2 (data not shown). Previously, we reported that murine CBA rAML cases without *Sfpi1/PU.1* involvement are rare (<10%; either as chromosome 2 deletion or as *Sfpi1/PU.1* exon 5 mutation), and that within this subset of rAMLs, ~50% of cases has internal tandem duplications (ITDs) within FMS-like tyrosine kinase 3 (*Flt3*) [23]. We have now screened a panel of 134 murine rAML samples and found *Flt3*-ITDs in 3% of the cases, none of them carrying deletions or mutations of *Sfpi1/PU.1*. Interestingly, 2.2% of cases carried a *KRAS* G12 mutation (manuscript in preparation).

### Simultaneous expansion of competing preleukemic clones

**Fig. 4:**
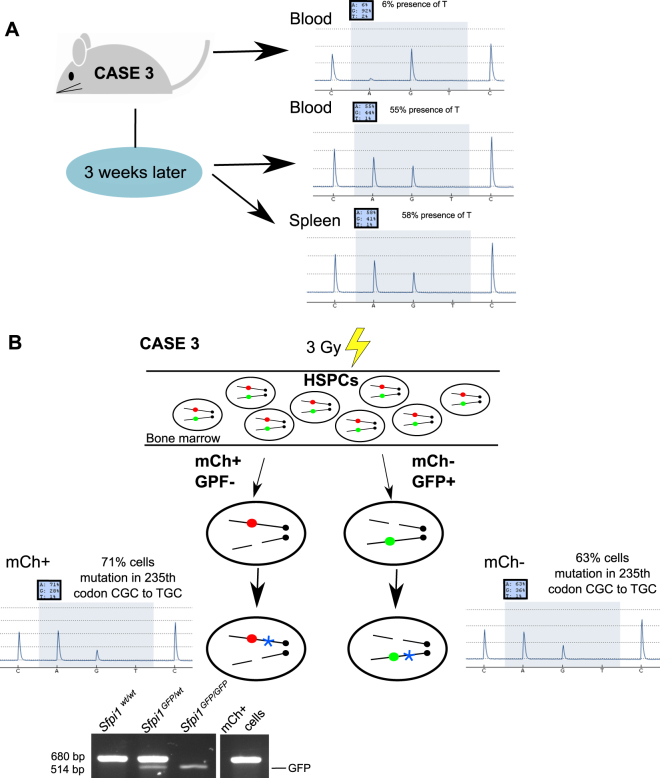
Simultaneous expansion of competing preleukemic clones. **a** Blood data of male mouse case 3 with mCherry− myeloid and lymphoid leukocytes. The mutation in the 235th codon of the *Sfpi1* gene (CGC to TGC) was analyzed at 15 months, and 21 days later at time of sacrifice, by pyrosequencing of blood and spleen. **b** Schematic representation of simultaneous clonal expansion of two preleukemic clones with loss of *Sfpi1* alleles (one with GFP and one with mCherry) in this leukemia case. The mutation in the *Sfpi1* gene was analyzed in mCherry+Lin− and mCherry−Lin− cells. Pyrograms for allele quantification for the first base of the 235th codon of the *Sfpi1* gene are presented for mCherry+Lin− and mCherry−Lin− cells. The presence of GFP in mCherry+Lin− cells was analyzed by PCR. A blue star depicts the presence of the mutation in the 235th codon of the Sfpi1 gene (CGC to TGC), red circle indicates mCherry and green circle GFP

At 15 months, one male mouse presented with an unusual clonal expansion of both mCherry−/GFP+ and mCherry+/GFP− leukocytes, with the point mutation detected in blood leukocytes (6%; Fig. 4a). Three weeks later, the point mutation had increased to 55% in blood (58% in spleen; Fig. 4a) and mCherry−Lin−/mCherry+Lin− cells were sorted. Despite being asymptomatic at sacrifice, this mouse had leukemic pathological features, demonstrating that appearance of the point mutation in Del2 blood cells was indicative of a preleukemic mouse at least 1 month prior to AML presentation. Hence, the point mutation occurs late in the leukemogenic process, but presents a rapid increase. Furthermore, both mCherry−/GFP+ and mCherry+/GFP− leukocytes were detected. A schematic representation of simultaneous expansion of two preleukemic clones (one clone with GFP loss and one with mCherry loss) is presented for this leukemic case (Fig. 4b). *Sfpi1* mutation was analyzed in mCherry+Lin− and mCherry−Lin− cells, and most mCherry− and mCherry+ cells carried the mutation (63% and 71%, respectively); loss of the GFP allele in mCherry+Lin− cells was also confirmed (Fig. 4b).

### Dysregulated PU.1 expression upon leukemic progression

**Fig. 5:**
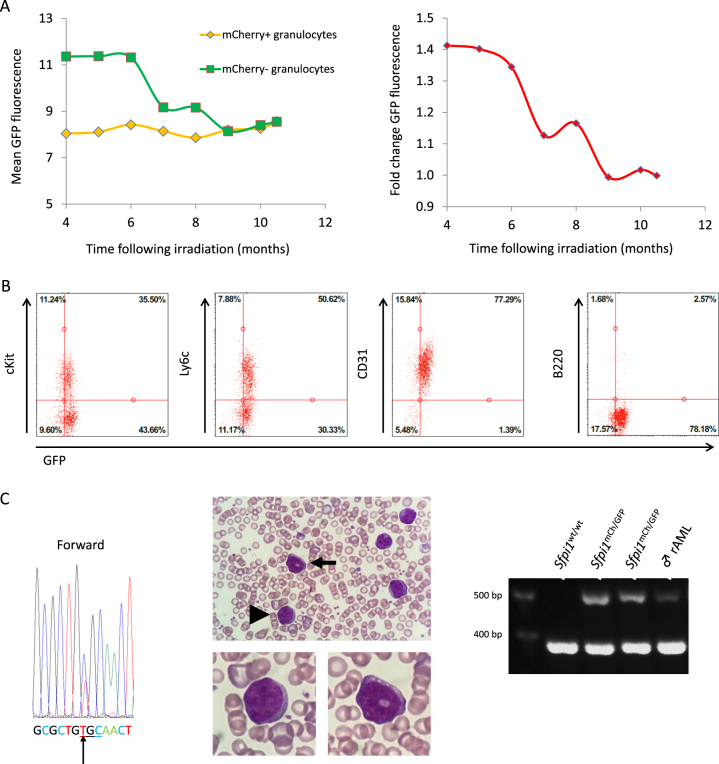
Leukemic progression following *Sfpi1* dysregulation. **a** At 6 months, GFP expression in mCherry− granulocytes of a male mouse had decreased slightly compared with mCherry− granulocytes at 4 months. GFP upregulation in mCherry− granulocytes decreased further, and at 9 months, GFP expression was no longer different. **b** mCherry−GFP+ spleen cells expressed immaturity cell surface markers. **c** Exon 5 *Sfpi1* DNA sequence of the leukemic mouse zoomed in on the CGC codon known to exhibit the point mutation (arrow indicates the point mutation). Blood smear showed nucleated cells twofold larger than RBCs with a blastic appearance. Confirmation of loss of mCherry allele: control DNA was obtained from a wild-type mouse and 2 sham-irradiated CBA *Sfpi1*^mCh/GFP^ mice [Rosa26-mCherry allele (510 bp) and wild-type allele (383 bp)]

We demonstrated that following Del2, PU.1 expression on the remaining homolog is upregulated, indicative of a negative feedback mechanism; with upregulation of the intact chr2 PU.1 promotor being a counter-balancing mechanism. However, in several cases, this feedback mechanism became dysregulated, possibly when the point mutation occurs, resulting in leukemia development. At 6 months, GFP expression of mCherry− granulocytes slightly decreased compared with mCherry+granulocytes (Fig. 5a). By 9 months, GFP expression was no longer increased. One month later, mCherry− leukocytes percentage had increased further. Shortly thereafter, the mouse presented with outward physical signs of rAML. Although still expressing GFP, 93.5% of cells were mCherry−, indicating Rosa26-mCherry chr2 homolog loss. Spleen cell immunophenotyping showed that mCherry−GFP+ cells expressed immaturity cell surface markers (Fig. 5b). Isolated DNA from these cells revealed a TGC sequence. Blood smear showed nucleated cells with blastic appearance while leukemic spleen cells had lost Rosa26-mCherry construct (Fig. 5c).

Notably, most female mice had clonal mCherry− lymphocytes expansion. Blood analysis of a female mouse showed that myeloid leukocytes retained mCherry expression at 13 months but 1.9% of lymphocytes were mCherry−, increasing further over time. At 17 months, the mouse presented with AML. All mCherry− leukemic spleen cells had lost mCherry and GFP expression, indicative of a lymphoid origin. Immunophenotyping showed that cells only expressed CD31 and B220 (Supplementary Figure S4A), and no mutation was found on the remaining GFP carrying chr2 homolog. On blood smear, most cells had round nuclei and high nucleus to cytoplasm ratio (Supplementary Figure S4B). Loss of Rosa26-mCherry construct was confirmed. While leukemic cells did not express GFP (i.e., PU.1 downregulation upon commitment towards the lymphoid lineage), the GFP construct was still detectable by PCR (Supplementary Figure S4C). Seven of the eight female mice were diagnosed with lymphocytic leukemia, while the remaining female was diagnosed with myeloid leukemia, with a much shorter latency compared with male AML cases (5 months and 11 months, respectively).

## Discussion

Using an engineered mouse model, we assessed Del2 occurrence after radiation exposure. A significant number of studies into the cytogenetics of rAML in various inbred mouse strains [24–26] revealed that structurally abnormal chr2—usually consisting of large hemizygous interstitial deletion (i.e., Del2)—occurs in ~90% of rAML cases, and typically is detectable in 90–100% of the leukemic cells within any sample [27]. It represents by far the most common and consistent chromosomal aberration seen in rAML mouse models. Of the other cytogenetic abnormalities identified in these studies, change in chromosome number is the most frequent. In particular, loss or gain in Y chromosome. More recently, we performed array comparative genomic hybridization (aCGH) at unprecedentedly high resolution on a unique panel of 79 CBA rAMLs [27]. Besides the characteristic Del2, small deletions were observed on chrs 3, 4, 5, 6, 11, and 16 in individual cases, but importantly no consistent event was identified.

At 9 months after radiation exposure, 25% of males presented with mCherry− leukocytes, in line with previously reported percentages in irradiated mice with BM Del2 clones, detected with cytogenetic methods [14]. All these male mice had mCherry loss in both myeloid and lymphoid lineages, indicative of a primitive hematopoietic cell type (HSC or MPP1). Using SLAM markers, we identified mCherry− HSCs (LSK CD48−CD150+) [28] in mice with mixed lineage mCherry− clonal expansion in blood. These mice had mCherry− myeloid and lymphoid progenitors, based on differential CD127 expression in these cells [29, 30], indicating that Del2 HSCs gave rise to mCherry− daughter HSCs as well as mCherry− lineage-committed progeny. At 15 months, 8 out of 18 males presented with lymphoid lineage-only mCherry− cells, indicating that in these mice Del2 likely occurred in a hematopoietic cell-like a CLP giving rise to lymphoid progeny specifically.

We assessed PU.1 expression from the remaining chr2 homolog in mice with mCherry− leukocytes (GFP as reporter for PU.1 transcription) [27, 31]. GFP expression in mCherry− myelocytes was 1.4-fold higher compared with mCherry+myelocytes. It can be directly attributed to an increased PU.1 promoter activity through PU.1 autoregulation to compensate the loss of the second allele. It was previously reported that both URE and *Sfpi1* proximal promoter have binding sites for PU.1 itself [32–34]. Here we report that during leukemogenesis, autoregulation of PU.1 was observed in mCherry− Lin− cells. This autoregulation becomes dysregulated, resulting in GFP expression decreasing to the level of mCherry+leukocytes. In these cases, immunophenotyping showed immature phenotypes consistent with myeloid leukemia. We also confirmed that the remaining *Sfpi1* copy carried a point mutation replacing arginine 235 with cysteine (the most common C to T substitution in murine rAMLs) [35] probably impairing transcriptional autoregulation. Our experimental observations are consistent with a two-hit model of murine radiation leukemogenesis in which the first irreversible mutational hit (deletion of one *Sfpi1* copy) results in preleukemic cells with growth advantage. Subsequently, they acquire a second mutational hit (point mutation in the remaining *Sfpi1*), leading to full malignancy, clonal expansion and leukemia [13].

It could be hypothesized that the point mutation occurs when the GFP levels return to ‘normal’ levels (i.e., equal to mCherry+ leukocytes), linking point mutation occurrence with loss of PU.1 autoregulation. Data obtained from terminal blood samples of irradiated mice revealed that 83% of mice had mutated cells. In contrast, the point mutation couldn’t be detected neither in sham-irradiated mice nor irradiated mice without mCherry− leukocytes. In addition, point mutations were exclusively detected in the BM mCherry−Lin− cell fraction of mice with mCherry− leukocytes. Most importantly, these observations link, for the first time, the occurrence of the point mutation to a prior presence of Del2. The leukemic case described with two competing preleukemic clones provides evidence of how rapid the leukemic progression occurs following acquisition of the second hit (6% to 55% in merely three weeks). To the best of our knowledge, only one study so far reported an alternative mutation occurring in a mutually exclusive way with *Sfpi1/PU.1* [23]. RFLP and sequencing analysis revealed in a subset of rAMLs the presence of *Flt3*-ITDs (one of the most frequent mutations in human AMLs [36]). These ITDs are similar to those seen in human AML cases and the mutual exclusion with *Sfpi1/PU.1* mutations suggests that *Flt3* mutations are driver mutations in these rare rAML cases. We have now screened a panel of 134 CBA rAML samples and found *Flt3*-ITDs in 3% of the cases (unpublished data). Although this is clearly a minor pathway, it represents the only significant alternative pathway to rAML identified so far in this mouse model with a direct link to human AML. In addition, we recently found a few rAML with leukemic cells carrying a *KRAS* mutation concomitantly to Del2 and *Sfpi1* mutations (data not shown), none of them with additional *Flt3*-ITDs, suggesting different leukemogenic pathways.

Overall male rAML incidence was 9% with an average latency of 11 months, consistent with previous work in male CBA mice [8, 16, 37]. One in three females had mCherry− lymphoid but not myeloid leukocytes at 12 months. The mean leukemic latency was similar to males (i.e., 13 and 11 months, respectively; *P* = 0.49). All but one male leukemia cases were categorized as myeloid leukemia. In contrast, 7 out of 8 female leukemia cases were categorized as lymphoid leukemia. Previous work reported AML incidences higher among irradiated males than females. [16, 38–40] Our data suggest that a gender-specific hematopoietic cell subpopulation leads to clonal expansion (HSC/MPP1 vs CLP). Interestingly, PU.1*-*knockout in lymphoid progenitors does not alter B-cell-maturation or proliferation [41], possibly explaining the mature B-cell phenotype in our female AMLs (i.e., B220 expression). On the other hand, PU.1-knockout in myeloid progenitors inhibits their maturation but not their proliferation [41], consistent with the immature myeloid phenotype observed in our male AMLs. However, we assume that all lineages are prone to Del2 to the same extent, but that cell intrinsic or external factors promoting their expansion, may be gender-specific.

An intrinsic gender-specific radiosensitivity is highly unlikely, and albeit not yet well understood, sex hormones might have a pivotal role. For example, castration does not affect rAML incidence, whereas ovariectomy results in a twofold increase [42]. Interestingly, gonadectomy does not alter leukemia incidence in unexposed mice [42]. It was also shown that estrogen binding to its receptor enhanced HSC self-renewal by upregulation of cell-cycle genes [43]. Follow-up of atomic bomb survivors reported that female AML baseline rate is ~40% of that for men who displayed a more rapid increased AML incidence rate with attained age [1]. In conclusion, we propose, based on our experimental data, a model with gender dependent leukemic pathways (Supplementary Figure S5). Our study provides novel insights into (radiation) leukemogenesis, and the model should enable further deciphering of this complex multistep process.

## Electronic supplementary material

Supplementary Figure Legends

Supplemental Figure S1

Supplemental Figure S2

Supplemental Figure S3

Supplemental Figure S4

Supplemental Figure S5
