# Supplementary material for: Tracking preleukemic cells in vivo to reveal the sequence of molecular events in radiation leukemogenesis
Source: Leukemia. 2018 Mar 3;32(6):1435–44. doi: 10.1038/s41375-018-0085-1 (PMC5990525; doi:10.1038/s41375-018-0085-1)
Supplement: Supplementary file 1 — Supplementary Figure Legends [file 41375_2018_85_MOESM1_ESM.docx]

**Supplementary Figure Legends**

**Supplementary Figure S1.** (A) Blood of irradiated male and female mice (n=70 and n=50, respectively) was analyzed monthly for mCherry and GFP expression. (B) Clonal expansion as a function of time observed in the fourteen female mice which presented with lymphoid clonal expansion at 15 months following irradiation (one line = one mouse).

**Supplementary Figure S2. Clonal expansion of mCherry- hematopoietic cells in bone marrow of irradiated male mice.**

Three male mice were sacrificed upon detection of clonal expansion. (A) Within the mCherry- LSK fraction, 54.7% of cells were CD48-CD150+. (B) CD127 expression in mCherry- and mCherry+ Lin- cells. (C) One mouse had no CD127 expression in mCherry-Lin- cells, consistent with the myeloid-biased phenotype observed in its peripheral blood.

**Supplementary Figure S3.** **Clonal expansion of mCherry- hematopoietic cells in bone marrow of irradiated female mice.**

Two female mice were sacrificed upon detection of lymphoid clonal expansion. (A) Within the mCherry- LSK fraction, most cells were Sca1-cKit- (95.7%). (B) CD127 expression in mCherry- and mCherry+ Lin- cells (left and right panel, respectively). Only mCherry- lymphocytes were observed in the peripheral blood.

**Supplementary Figure S4. Leukemic progression in a female irradiated CBA *Sfpi1*^mCh/GFP^ mouse.** (A) Cell surface marker expression of spleen cells. (B) Blood smear showed nucleated cells two-fold larger than RBCs with a homogeneous appearance. (C) PCRs were performed amplifying the region containing the mCherry or GFP construct [left and right panel, respectively]. GFP allele (512 bp) and wild-type allele (680 bp).

**Supplementary Figure S5. Model of the different leukemic pathways observed in CBA *Sfpi1*^mCh/GFP^ mice.**
