# Supplementary material for: Tracking preleukemic cells in vivo to reveal the sequence of molecular events in radiation leukemogenesis
Source: Leukemia. 2018 Mar 3;32(6):1435–44. doi: 10.1038/s41375-018-0085-1 (PMC5990525; doi:10.1038/s41375-018-0085-1)
Supplement: Supplementary file 2 — Supplemental Figure S1 [file 41375_2018_85_MOESM2_ESM.pptx]

## Slide 1
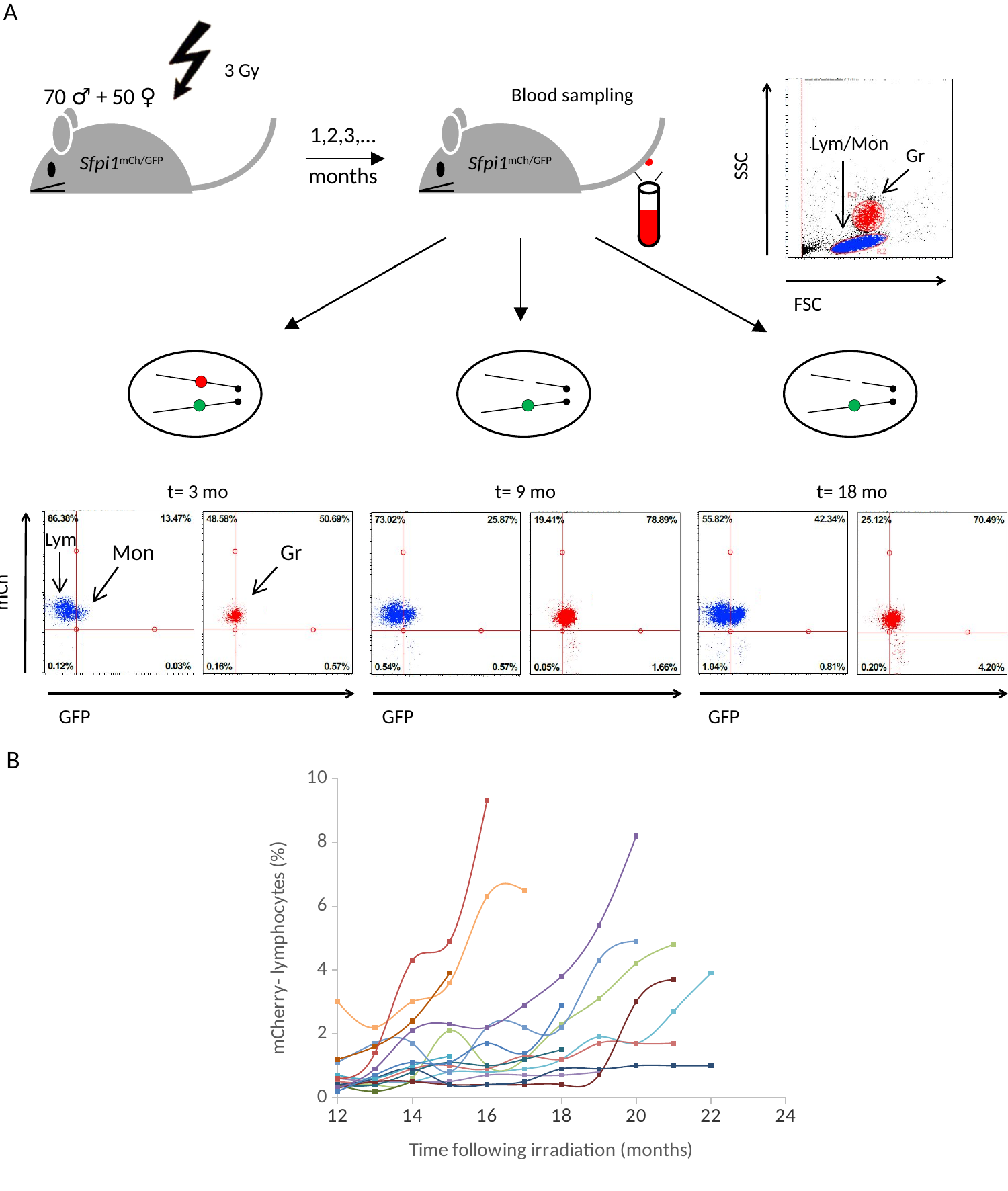

A
Sfpi1mCh/GFP
3 Gy
70 ♂ + 50 ♀
Blood sampling
SSC
1,2,3,…
months
Lym/Mon
Gr
Sfpi1mCh/GFP
FSC
t= 3 mo
t= 18 mo
t= 9 mo
mCh
 Lym
Mon
Gr
GFP
GFP
GFP
B
### Chart
| Category | MCh 32.2 d (10) | MCh 32.2 b (1) | MCh 30.2 (11) | MCh 31.1 (13) | MCh 24.2 d (31) | MCh 24.2 b (11) | MCh 13.5 c (13) | MCh 13.4 b (11) | MCh 13.4 a (8) | MCh 25.1 b (11) | MCh 25.1 a (8) | MCh 17.2 b (11) | MCh 13.1 c (13) | MCh 15.1 b (11) | MCh 5.2 a (8) |
|---|---|---|---|---|---|---|---|---|---|---|---|---|---|---|---|
