# Supplementary material for: Tracking preleukemic cells in vivo to reveal the sequence of molecular events in radiation leukemogenesis
Source: Leukemia. 2018 Mar 3;32(6):1435–44. doi: 10.1038/s41375-018-0085-1 (PMC5990525; doi:10.1038/s41375-018-0085-1)
Supplement: Supplementary file 4 — Supplemental Figure S3 [file 41375_2018_85_MOESM4_ESM.pptx]

## Slide 1
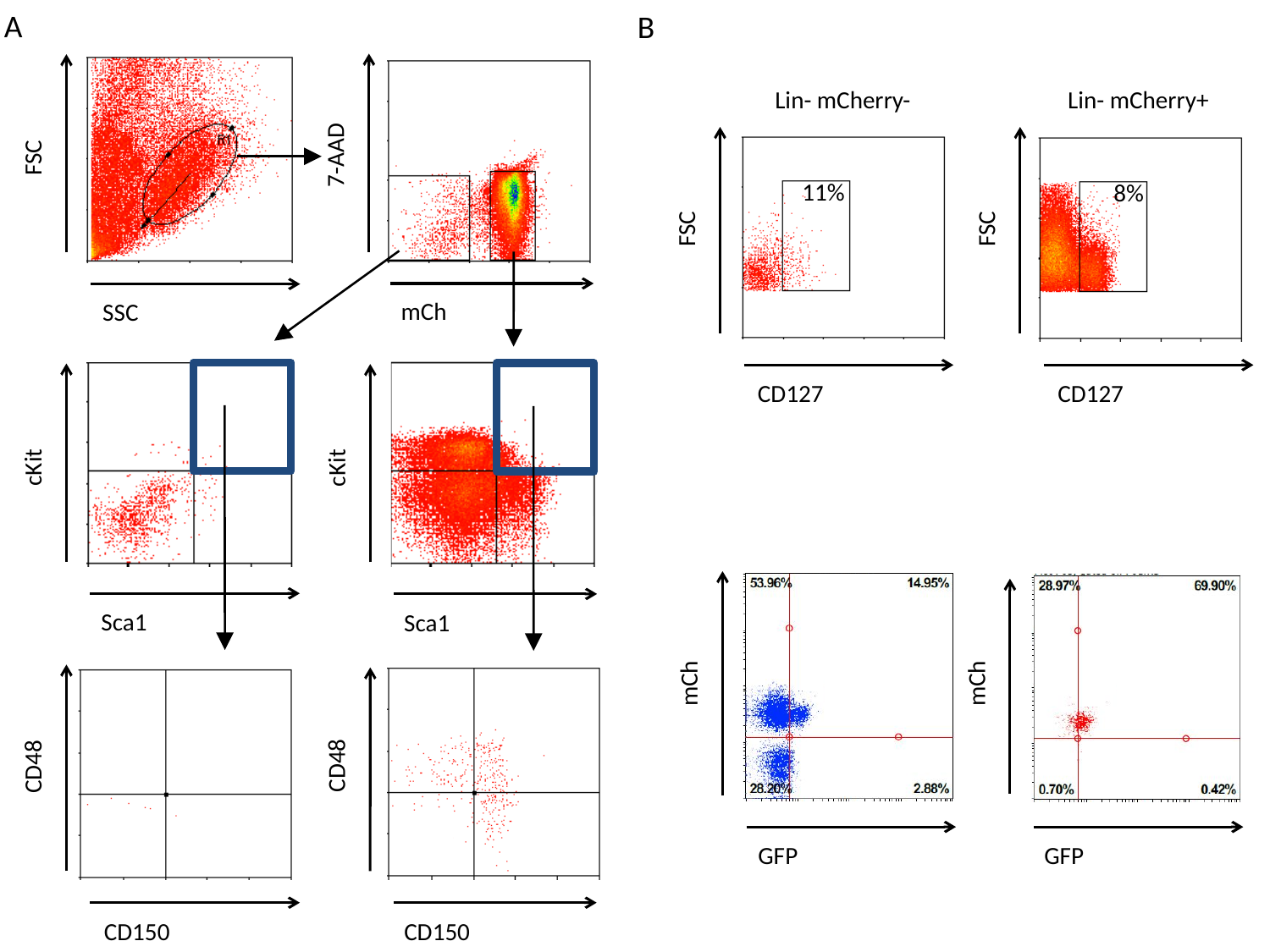

A
B
7-AAD
FSC
Lin- mCherry-
Lin- mCherry+
FSC
FSC
11%
8%
mCh
SSC
CD127
CD127
cKit
cKit
mCh
mCh
Sca1
Sca1
CD48
CD48
GFP
GFP
CD150
CD150
