# Supplementary material for: Tracking preleukemic cells in vivo to reveal the sequence of molecular events in radiation leukemogenesis
Source: Leukemia. 2018 Mar 3;32(6):1435–44. doi: 10.1038/s41375-018-0085-1 (PMC5990525; doi:10.1038/s41375-018-0085-1)
Supplement: Supplementary file 5 — Supplemental Figure S4 [file 41375_2018_85_MOESM5_ESM.pptx]

## Slide 1
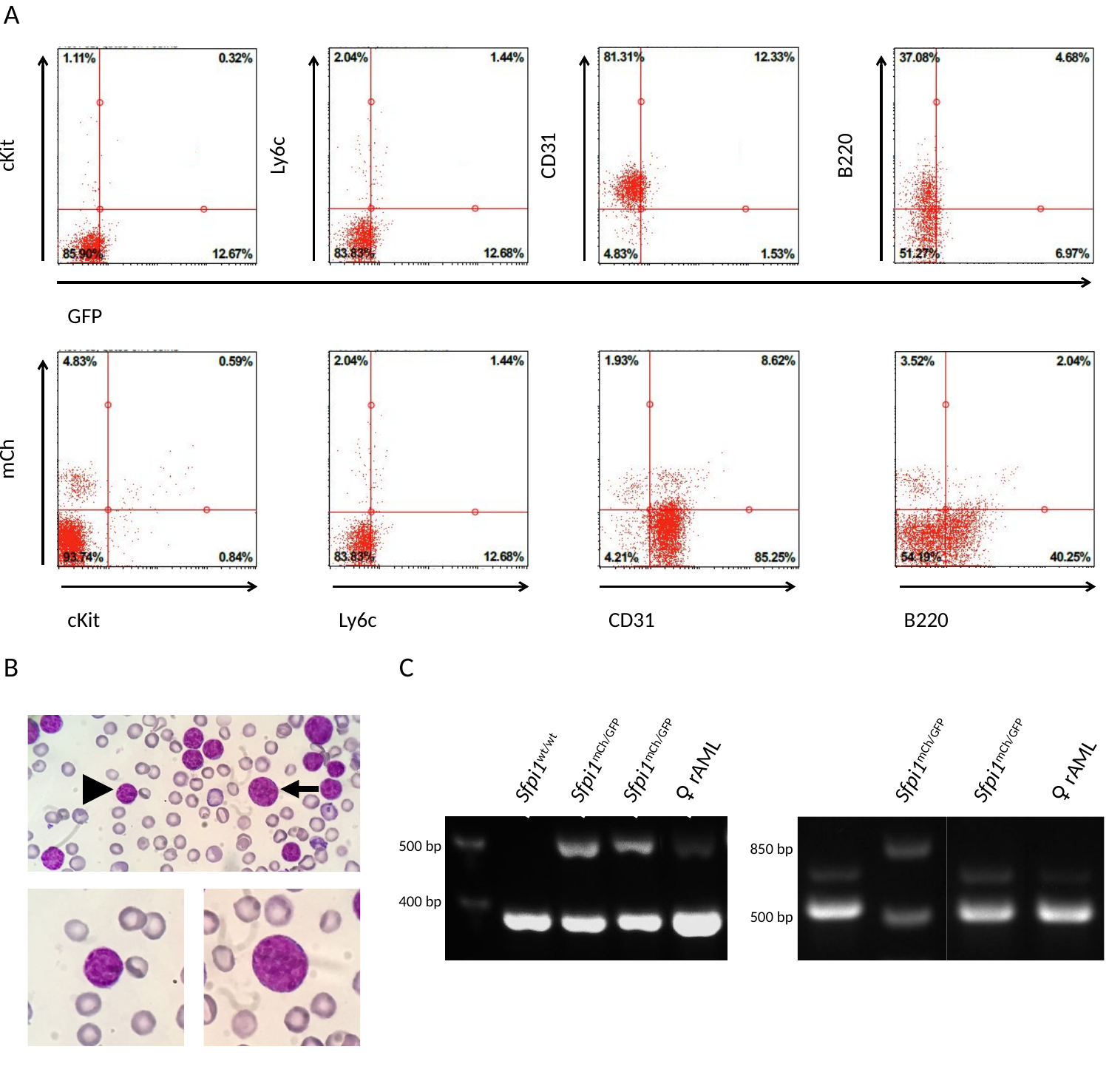

A
cKit
Ly6c
CD31
B220
GFP
mCh
cKit
Ly6c
CD31
B220
B
C
♀ rAML
Sfpi1mCh/GFP
♀ rAML
Sfpi1mCh/GFP
Sfpi1mCh/GFP
Sfpi1wt/wt
Sfpi1mCh/GFP
500 bp
400 bp
850 bp
500 bp
