# Supplementary figures and images for: Tracking preleukemic cells in vivo to reveal the sequence of molecular events in radiation leukemogenesis
Source: Leukemia. 2018 Mar 3;32(6):1435–44. doi: 10.1038/s41375-018-0085-1 (PMC5990525; doi:10.1038/s41375-018-0085-1)

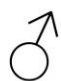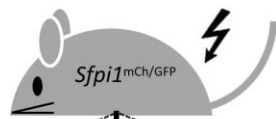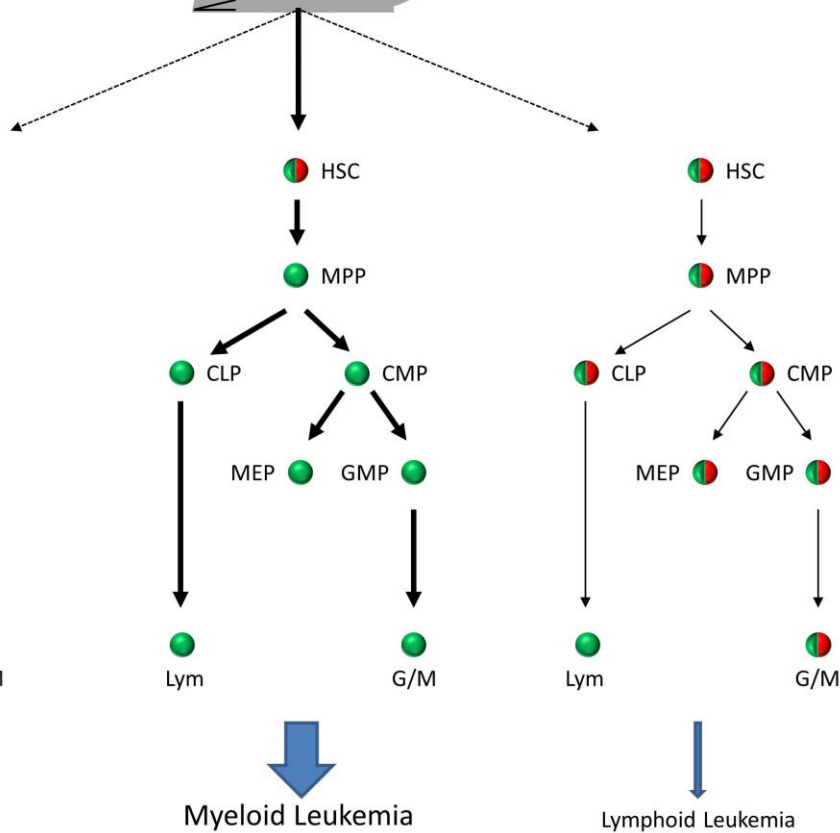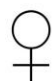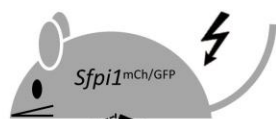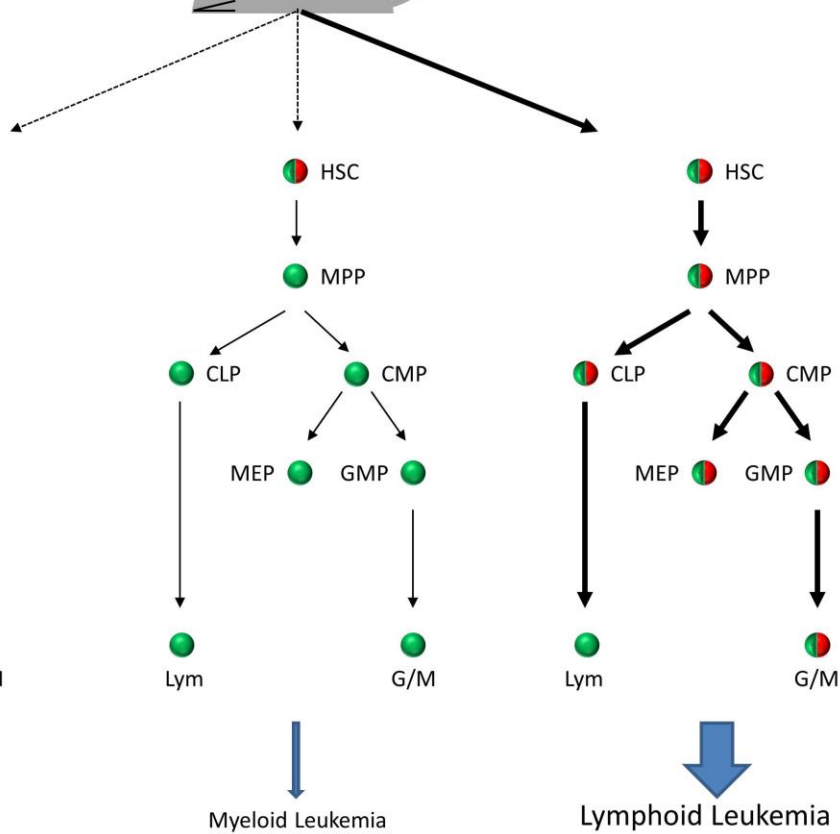

Supplement: Supplementary file 6 — Supplemental Figure S5 [file 41375_2018_85_MOESM6_ESM.pdf]
